# Pain and Its Association with Survival for Black and White Individuals with Advanced Prostate Cancer in the United States

**DOI:** 10.1158/2767-9764.CRC-23-0446

**Published:** 2024-01-08

**Authors:** Emily M. Rencsok, Natalie Slopen, Hannah D. McManus, Karen A. Autio, Alicia K. Morgans, Lawrence McSwain, Pedro Barata, Heather H. Cheng, Robert Dreicer, Travis Gerke, Rebecca Green, Elisabeth I. Heath, Lauren E. Howard, Rana R. McKay, Joel Nowak, Shannon Pileggi, Mark M. Pomerantz, Dana E. Rathkopf, Scott T. Tagawa, Young E. Whang, Camille Ragin, Folakemi T. Odedina, Philip W. Kantoff, Jake Vinson, Paul Villanti, Sebastien Haneuse, Lorelei A. Mucci, Daniel J. George, for the IRONMAN Registry

**Affiliations:** 1 Department of Epidemiology, Harvard T.H. Chan School of Public Health, Boston, Massachusetts.; 2 Harvard-MIT Division of Health Sciences and Technology, Harvard Medical School, Boston, Massachusetts.; 3 Department of Social and Behavioral Sciences, Harvard T.H. Chan School of Public Health, Boston, Massachusetts.; 4 Duke Cancer Institute, Durham, North Carolina.; 5 Memorial Sloan Kettering Cancer Center, New York, New York.; 6 Dana-Farber Cancer Institute, Boston, Massachusetts.; 7 Patient author, Durham, North Carolina.; 8 Section of Hematology and Oncology, Tulane University School of Medicine, New Orleans, Louisiana.; 9 University Hospitals Seidman Cancer Center, Cleveland, Ohio.; 10 Division of Medical Oncology, University of Washington, Seattle, Washington.; 11 Fred Hutchinson Cancer Center, Seattle, Washington.; 12 University of Virginia Cancer Center, Charlottesville, Virginia.; 13 Prostate Cancer Clinical Trials Consortium (PCCTC), New York, New York.; 14 Karmanos Cancer Institute, Detroit, Michigan.; 15 Department of Oncology, University of California San Diego Moores Cancer Center, La Jolla, California.; 16 Cancer ABCs, Brooklyn, New York.; 17 Division of Hematology and Medical Oncology, Weill Cornell Medical Center, New York, New York.; 18 Department of Medicine, Division of Oncology, University of North Carolina School of Medicine, Chapel Hill, North Carolina.; 19 Fox Chase Cancer Center, Philadelphia, Pennsylvania.; 20 African-Caribbean Cancer Consortium, Philadelphia, Pennsylvania.; 21 Mayo Clinic Comprehensive Cancer Center, Jacksonville, Florida.; 22 Prostate Cancer Transatlantic Consortium (CaPTC), Jacksonville, Florida.; 23 Convergent Therapeutics, Cambridge, Massachusetts.; 24 Movember Foundation, Melbourne, Australia.; 25 Department of Biostatistics, Harvard T.H. Chan School of Public Health, Boston, Massachusetts.

## Abstract

Bone pain is a well-known quality-of-life detriment for individuals with prostate cancer and is associated with survival. This study expands previous work into racial differences in multiple patient-reported dimensions of pain and the association between baseline and longitudinal pain and mortality. This is a prospective cohort study of individuals with newly diagnosed advanced prostate cancer enrolled in the International Registry for Men with Advanced Prostate Cancer (IRONMAN) from 2017 to 2023 at U.S. sites. Differences in four pain scores at study enrollment by race were investigated. Cox proportional hazards models and joint longitudinal survival models were fit for each of the scale scores to estimate HRs and 95% confidence intervals (CI) for the association with all-cause mortality. The cohort included 879 individuals (20% self-identifying as Black) enrolled at 38 U.S. sites. Black participants had worse pain at baseline compared with White participants, most notably a higher average pain rating (mean 3.1 vs. 2.2 on a 10-point scale). For each pain scale, higher pain was associated with higher mortality after adjusting for measures of disease burden, particularly for severe bone pain compared with no pain (HR, 2.47; 95% CI: 1.44–4.22). The association between pain and all-cause mortality was stronger for participants with castration-resistant prostate cancer compared with those with metastatic hormone-sensitive prostate cancer and was similar among Black and White participants. Overall, Black participants reported worse pain than White participants, and more severe pain was associated with higher mortality independent of clinical covariates for all pain scales.

**Significance:**

Black participants with advanced prostate cancer reported worse pain than White participants, and more pain was associated with worse survival. More holistic clinical assessments of pain in this population are needed to determine the factors upon which to intervene to improve quality of life and survivorship, particularly for Black individuals.

## Introduction

In the United States, an estimated 120,000 individuals are living with advanced prostate cancer (1), defined as metastatic hormone-sensitive prostate cancer (mHSPC) or castration-resistant prostate cancer (CRPC; ref. 2). Black individuals experience the most significant advanced prostate cancer burden with over double the age-adjusted prevalence and mortality compared with White individuals (1).

An important quality-of-life detriment experienced by many individuals with advanced prostate cancer is pain, often driven by metastasis to the bone (3). In addition to bone pain, other types of pain, including neuropathic, nociceptive, osteoarthritic, and muscular pain, are frequently experienced as chronic pain with poorly-managed breakthrough episodes (4). Pain is a subjective experience shaped by cultural and individual expectations in addition to exacerbating factors such as psychosocial stressors, medical comorbidities, and ability to navigate the health system (5).

In this study, we consider race a social construct that serves as a proxy marker for a range of social experiences shaped by structural racism (6). As a result of structural racism, social determinants of health impact the lives of Black individuals in almost every domain of life (health, education, housing, employment, etc.). The subjective experience of pain described above can also be shaped by these social determinants of health, leading to differential reporting and experience of pain by race. Importantly, individual and systemic discrimination can also lead to health professionals prescribing analgesics less frequently to Black and Hispanic patients with cancer compared with White patients, further exacerbating disparities in the experience and management of pain in health settings (7).

Previous studies have shown that Black individuals with advanced prostate cancer report more severe bone pain than White individuals (8, 9). In addition, our group has previously shown that Black participants in the International Registry for Men with Advanced Prostate Cancer (IRONMAN) registry reported worse pain overall at study enrollment compared with White participants, and that pain increased throughout the first year of follow-up similarly for both racial groups (10). While these differences in bone and overall pain by race are known, it is not clear whether various aspects of pain (severity, duration, interference with daily activities, etc.) differ by race. This is important to investigate as pain is notoriously difficult to both report and interpret, particularly when interpersonal relationships and other social determinants of health affect the perception of the scale used for both the patient and the health professional (11). More holistically assessing the various dimensions of pain can better facilitate communication about the patient's experience of pain, leading to more cultural sensitivity around perceptions of pain and best methods for management (12).

Increased pain overall has been shown to be associated with worse survival in advanced prostate cancer populations (13–16); however, most studies were conducted in primarily White populations participating in randomized controlled trials of disease-directed therapies. There is a need to expand this research into a more real-world population, inclusive of non-White individuals and also individuals who have additional medical or socioeconomic considerations leading them to be unable to participate in randomized controlled trials.

This study aims to descriptively assess multidimensional pain (pain interference, average pain, worst pain, and bone pain) in Black and White individuals with newly diagnosed mHSPC or CRPC participating in the IRONMAN registry to better describe the experience of pain in this patient population, laying the groundwork for future studies to investigate potential mediators of the race-pain relationship (e.g., access to care, analgesic prescription, etc.) and interventions to improve quality of life. We also investigate the association between each baseline and longitudinal pain scale and survival in the overall study population to give insight into which aspects of pain have the largest prognostic value and should be followed up most closely during health appointments.

We hypothesize that Black participants will report worse pain across all scales since Black individuals tend to have more advanced prostate cancer at diagnosis and social determinants that more negatively impact their health compared with White individuals. We additionally hypothesize that interference of pain with daily activities and bone pain will have the greatest prognostic value for overall survival as they are most representative of the impact of pain on the patient's life and also likely of disease burden due to metastases to the bone.

## Materials and Methods

### Study Participants

Study participants included individuals newly diagnosed with mHSPC or CRPC who enrolled in the IRONMAN registry (NCT 03151629) between July 21, 2017 and January 23, 2023. IRONMAN is an international prospective cohort of individuals with no more than 90 days of life-prolonging therapy prior to enrollment for patients with CRPC and no more than 90 days of active therapy including androgen deprivation therapy (ADT) for patients with mHSPC. Participants are recruited through IRONMAN-affiliated clinicians in 16 countries (17). Study sites are primarily located in urban centers in regions with high prostate cancer mortality. This analysis focused on individuals enrolled in the United States. All study participants gave written informed consent prior to study enrollment and were able to withdraw from the study at any time. This study was approved by the Harvard Longwood Campus Institutional Review Board guided by the ethical principles set forth in the Belmont Report.

### Exposure Measures: Pain

Pain was self-reported by study participants at enrollment and every 3–6 months throughout a follow-up period of up to 5 years (17). The European Organization for Research and Treatment of Cancer Quality of Life Questionnaire (EORTC QLQ-C30) contains two questions on the presence and interference of pain rated on a Likert scale of 1 (“not at all”) to 4 (“very much”). These two questions are combined and linearly transformed to a pain scale score with a range of 0 (least severe) to 100 (most severe; ref. 18). The minimally important difference (MID) for deterioration on the EORTC pain scale for individuals with prostate cancer is 5 points (19). The EORTC QLQ-C30 pain scale has shown high reliability (Cronbach alpha 0.83) in a racially diverse population over the age of 50 years (20).

The Brief Pain Inventory (BPI) contains two questions on severity of average and worst pain in the past 24 hours rated on a scale of 1 (“no pain”) to 10 (“pain as bad as you can imagine”). The MID for deterioration on the BPI scale is between 1.3 and 1.6 points (21). The BPI has shown high construct validity in a racially diverse population and high concordance with other pain scales in an advanced prostate cancer population (22, 23).

The Functional Assessment of Cancer Therapy Prostate Cancer Symptom Index (FACT-FPSI) contains one question on presence of bone pain in the past week rated on a Likert scale of 0 (“not at all”) to 4 (“very much”). While the MID and reliability/validity for the bone pain question have not specifically been investigated, these have been assessed for the overall FACT-FPSI scale in patients with prostate cancer (24, 25).

### Demographic and Clinical Characteristics

Demographic information (age, highest level of education, employment status, marital status, military status, race, ethnicity) was collected through patient-reported questionnaires at study enrollment. Clinical variables (disease state at enrollment, Gleason score, first on-study PSA level, treatments received prior to study and throughout follow-up, metastatic status at study enrollment, *de novo* metastases at study enrollment, sites of metastases at study enrollment, and type of health center) were abstracted from patient medical records and entered by study sites at the time of enrollment. Gleason score was assessed from prostate biopsy, radical prostatectomy, transurethral resection of the prostate (TURP), or biopsy of a metastatic site at time of prostate cancer diagnosis or surgical treatment. Disease state was categorized as mHSPC (*de novo* metastatic disease at diagnosis or progressed to metastasis after localized prostate cancer diagnosis) and M0 or M1 CRPC (progression of disease while on ADT or with castrate level of testosterone determined by the investigator).

### Statistical Analysis

We summarized demographic and clinical variables stratified by race at study enrollment. Next, we investigated racial differences in the four pain scales at enrollment by comparing average scores with *t* tests and *χ*^2^ tests as appropriate and creating histograms of all scores. We calculated the Pearson correlation between each of the pain scales to investigate whether the different scales measure different aspects of pain.

Second, we defined the time to all-cause mortality as the time from the date of study enrollment (referred to as “baseline” in this study) to the date of death and constructed Kaplan–Meier estimates of the survivor function to visualize differences by race, disease state, and pain scale score categories. We created three categories of pain for each pain scale corresponding to no pain, a little/light pain, and moderate-to-severe pain; significance of differences in survival by pain category were assessed using the log-rank test. We estimated 80th percentile survival and 95% confidence intervals (CI) for each category of pain at study enrollment; due to short follow-up for many participants, we were not able to estimate median overall survival.

Third, we fit separate Cox proportional hazards models using the imputed datasets to investigate the association between each of the four pain scales at enrollment and all-cause mortality. Missing individual covariates and pain scale scores on completed questionnaires were imputed using multiple imputation by chained equations (MICE; ref. 26) and sensitivity analyses were conducted to determine the impact of varying the missing indicator used. An in-depth description of missing data exploration and methods is included in Supplementary Data S1, and an overview of longitudinal missing data is shown in Supplementary Fig. S1. All Cox models were adjusted for potential confounders of disease burden including age at study enrollment (continuous), first PSA on study (continuous), Gleason score (categorical; 6 or less, 7, 8, or 9–10), disease state at enrollment (mHSPC or CRPC), *de novo* metastatic status at enrollment (yes/no), and site of metastases at baseline (categorical; none, lymph node only, bone and/or lymph node only, liver/lung metastases present, or other). Participants were clustered by site ID (*N* = 38) with robust SEs assuming there may be some correlation of pain scores within study site due to practice patterns, and the baseline hazard was stratified by year of enrollment. HRs and 95% CIs were estimated and pooled across imputed datasets using Rubin Rules (27). Analyses were additionally stratified by disease state at enrollment and race.

Finally, to investigate the association between longitudinal pain score trajectories throughout follow-up and mortality, we fit joint longitudinal survival models for each pain scale (28). Joint longitudinal survival models simultaneously fit two models: one model for the longitudinal trajectory of pain over time and one model for the multivariable-adjusted association between pain and all-cause mortality, ultimately estimating the averaged association between pain and survival given pain scores at each timepoint. For the models representing the longitudinal trajectories of pain, we fit linear mixed effects models for the pain scales with month of questionnaire as linear, quadratic, and cubic terms and with observations clustered within study participants. While we do not explicitly model the trajectories of pain in this study, our group has previously modeled the longitudinal trajectory of the EORTC pain scale in this cohort over the first year of follow-up (10).

Each linear mixed effects model was then jointly modeled with the same Cox models described above using the JM package in R, assuming a Weibull baseline hazard (29). Results were pooled across imputed datasets, and HRs and 95% CIs for the association between the time-varying pain scale scores and mortality were estimated; similar to a time-dependent Cox model, this HR represents the average of the HRs for the association of a one-point increase in pain at each timepoint with all-cause mortality. While the association between longitudinal pain interference, average pain, and worst pain with mortality could be estimated with the joint modeling procedure, the association between bone pain and mortality was not able to be assessed because of incompatibility of categorical longitudinal outcomes with joint modeling capabilities in the JM package. All analyses were completed using R version 4.1.0 with statistical significance assessed at the 0.05 level.

An advanced prostate cancer survivor is an author on this article, and a glossary of technical terms is included in Supplementary Data S1 accompanying this article for increased accessibility to non-academic audiences.

### Data Availability Statement

The data analyzed in this study are available from the Prostate Cancer Clinical Trials Consortium. Restrictions apply to the availability of these data, which were used under agreement for this study. Data are available from the authors upon reasonable request with the permission of the Prostate Cancer Clinical Trials Consortium.

## Results

### Participant Characteristics

This analysis included 879 participants from IRONMAN self-identifying as White (*N* = 704, 80%) or Black (*N* = 175, 20%) who received care at 38 study sites across the United States (Supplementary Fig. S2; Supplementary Table S1). Demographic and clinical characteristics stratified by self-reported race are shown in Table 1. For the entire cohort, the mean age at enrollment was 69.1 (SD 8.9 years), and a larger portion of the participants (65%) had mHSPC at enrollment compared with CRPC (35%). The most commonly received therapies at any point on study were ADT (89%), androgen receptor signaling inhibitors (ARSI; 67%), and chemotherapy (24%; Supplementary Table S2).

**TABLE 1:** Cohort demographic and clinical characteristics by self-reported race (*N* = 879), 2017–2023

	White (*N* = 704)	Black (*N* = 175)
Age at study entry, years		
Mean (SD)	69.5 (9.0)	67.2 (8.7)
Disease state at enrollment		
CRPC	241 (34%)	65 (37%)
mHSPC	463 (66%)	110 (63%)
*De novo* metastatic disease at enrollment		
Yes	294 (68%)	70 (71%)
No	137 (32%)	28 (29%)
Missing	*n* = 273	*n* = 77
Location of metastases at enrollment		
No metastases at baseline	29 (7%)	4 (3%)
Lymph nodes only	36 (9%)	15 (13%)
Bone ± lymph nodes only	245 (63%)	77 (66%)
Other soft-tissue metastases present	80 (21%)	21 (18%)
Missing	*n* = 314	*n* = 58
Prostatectomy or biopsy Gleason score		
6 or less	26 (5%)	3 (2%)
7	163 (28%)	45 (34%)
8	106 (18%)	20 (15%)
9–10	278 (49%)	63 (48%)
Missing	*n* = 131	*n* = 44
First on-study PSA (ng/mL)		
Mean (SD)	88.6 (484.8)	156.9 (396.3)
Missing	*n* = 33	*n* = 8
Hispanic/Latino		
No	657 (97%)	156 (96%)
Yes	22 (3%)	6 (4%)
Missing	*n* = 25	*n* = 13
Highest education level at baseline		
Less than College	29 (14%)	18 (43%)
Some College or Bachelor's degree	72 (35%)	13 (31%)
Vocational School/Program	2 (1%)	1 (2%)
Graduate degree	101 (49%)	9 (21%)
Other	3 (1%)	1 (2%)
Missing	*n* = 497	*n* = 133
Marital status at baseline		
Married	545 (78%)	88 (51%)
In a civil partnership	20 (3%)	2 (1%)
Widowed	29 (4%)	12 (7%)
Divorced/Separated	76 (11%)	43 (25%)
Never married	28 (4%)	26 (15%)
Missing	*n* = 6	*n* = 4
Employment status at baseline		
Retired	408 (58%)	82 (48%)
Working full-time	200 (29%)	45 (26%)
Working part-time	58 (8%)	11 (6%)
Unemployed	12 (2%)	16 (9%)
Disabled	22 (3%)	17 (10%)
Missing	*n* = 4	*n* = 4
Member of national military at baseline		
Yes, currently or previously	182 (33%)	37 (28%)
No, I have never served in the national military	364 (67%)	97 (72%)
Missing	*n* = 158	*n* = 41
Type of health center		
Clinic	30 (4%)	7 (4%)
Hospital	125 (18%)	21 (12%)
NCI-designated	535 (76%)	131 (75%)
VA	14 (2%)	16 (9%)
Time on study (months)		
Mean (SD)	28.9 (17.4)	24.8 (17.2)

Abbreviations: CRPC, castration-resistant prostate cancer; mHSPC, metastatic hormone-sensitive prostate cancer; PSA, prostate-specific antigen.

Overall, clinical disease characteristics were similar by race with the exception of a higher PSA level in Black participants compared with White participants (Table 1). Regarding demographic characteristics, Black participants were younger at study enrollment, reported lower education, were less likely to be married and were less likely to be retired compared with White participants.

### Baseline Differences in Pain by Self-reported Race

At study enrollment, Black participants reported more severe pain on average across all pain scales compared with White participants with the exception of bone pain, where no differences by race were seen (Table 2). Racial differences in each pain scale were similar for both participants with mHSPC and CRPC; for example, White participants with mHSPC had a mean EORTC pain scale score of 19.1 compared with the mean of 26.7 for Black participants with mHSPC. The scale scores for participants with CRPC were similar (mean 18.4 for White participants and 26.2 for Black participants).

**TABLE 2:** Average pain at study enrollment by disease state at enrollment and self-reported race (*N* = 4 pain scales)

	CRPC			mHSPC		
	White (*N* = 241)	Black (*N* = 65)	*P*-value	White (*N* = 463)	Black (*N* = 110)	*P*-value
EORTC QLQ-C30 Pain Scale (0–100)^a^ *Minimally important difference: 5 points*						
Mean (SD)	18.4 (23.1)	26.2 (28.1)	0.05	19.1 (25.0)	26.7 (30.0)	0.03
Missing	*n* = 33	*n* = 4		*n* = 67	*n* = 19	
Q1: How often have you had pain in the past week?						
1 – Not at all	108 (51%)	23 (38%)	0.13	200 (51%)	38 (41%)	0.09
2 – A little	77 (37%)	25 (41%)		138 (35%)	33 (36%)	
3 – Quite a bit	18 (9%)	8 (13%)		42 (11%)	12 (13%)	
4 – Very much	7 (3%)	5 (8%)		16 (4%)	9 (10%)	
Missing	*n* = 31	*n* = 4		*n* = 67	*n* = 18	
Q2: How often did pain interfere with your daily activities in the past week?						
1 – Not at all	132 (63%)	34 (56%)	0.26	265 (67%)	53 (58%)	0.11
2 – A little	58 (28%)	17 (28%)		96 (24%)	22 (24%)	
3 – Quite a bit	15 (7%)	7 (11%)		20 (5%)	8 (9%)	
4 – Very much	3 (1%)	3 (5%)		16 (4%)	8 (9%)	
Missing	*n* = 33	*n* = 4		*n* = 66	*n* = 19	
Brief Pain Inventory (BPI) *Minimally important difference: 1.3–1.6 points*						
Rate your average pain (1–10)						
Mean (SD)	2.2 (1.6)	3.1 (2.2)	0.01	2.2 (1.6)	3.2 (2.4)	<0.001
Missing	*n* = 42	*n* = 10		*n* = 73	*n* = 19	
Rate your pain at its worst in the last 24 hours (1–10)						
4 or greater	48 (24%)	18 (33%)	0.27	85 (22%)	37 (41%)	<0.001
Less than 4	151 (76%)	37 (67%)		305 (78%)	54 (59%)	
Missing	*n* = 42	*n* = 10		*n* = 73	*n* = 19	
FACT-FPSI *Minimally important difference: Not established*						
How often have you had bone pain in the past week?						
0 – Not at all	120 (60%)	31 (55%)	0.90	234 (61%)	49 (55%)	0.27
1 – A little bit	42 (21%)	14 (25%)		86 (22%)	21 (24%)	
2 – Somewhat	21 (11%)	6 (11%)		27 (7%)	5 (6%)	
3 – Quite a bit	10 (5%)	4 (7%)		31 (8%)	9 (10%)	
4 – Very much	6 (3%)	1 (2%)		7 (2%)	5 (6%)	
Missing	*n* = 42	*n* = 9		*n* = 78	*n* = 21	

Abbreviations: CRPC, castration-resistant prostate cancer; mHSPC, metastatic hormone-sensitive prostate cancer.

^a^The EORTC pain scale score is created through a linear transformation of Q1 and Q2 below.

**FIGURE 1:**
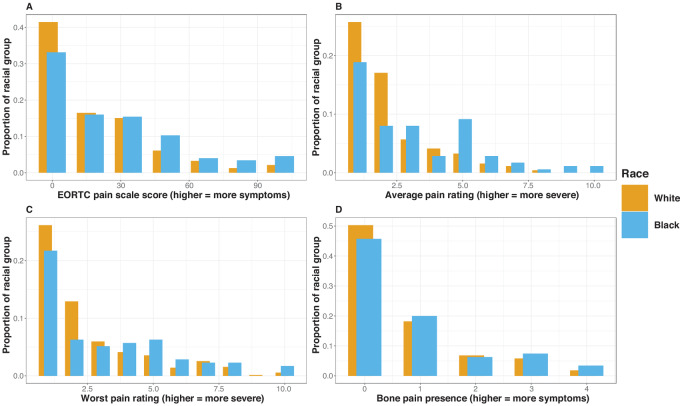
Distributions of pain at study enrollment by race. **A,** Distributions of scores on the EORTC pain scale by race. **B,** Distributions of scores on the average pain scale by race. **C,** Distributions of scores on the worst pain scale by race. **D,** Distributions of scores on the bone pain scale by race.

Though the largest proportion of participants reported no pain at baseline across each of the four pain scales, the majority of study participants reported at least some pain on each of the scales, with Black participants tending to report more severe pain (Fig. 1). The four pain scales have moderate-to-high correlation with each other (correlation coefficient between 0.56 and 0.87; Supplementary Table S3).

### Differences in Survival Time by Self-reported Race, Disease State, and Baseline Pain

The median follow-up time for White participants in the study was 2.24 years compared with 1.73 years for Black participants with 137 (19.5%) White participants and 37 (21.4%) Black participants dying throughout follow-up (Supplementary Table S4). The 80th percentile survival for White participants was 2.57 years (95% CI: 2.34–2.95) and for Black participants was 2.09 years (95% CI: 1.68–2.98; Supplementary Fig. S3). The 80th percentile survival for participants with mHSPC was 2.84 years (95% CI: 2.57–3.83) and for participants with CRPC was 2.08 years (95% CI: 1.75–2.40; Supplementary Fig. S4).

**FIGURE 2:**
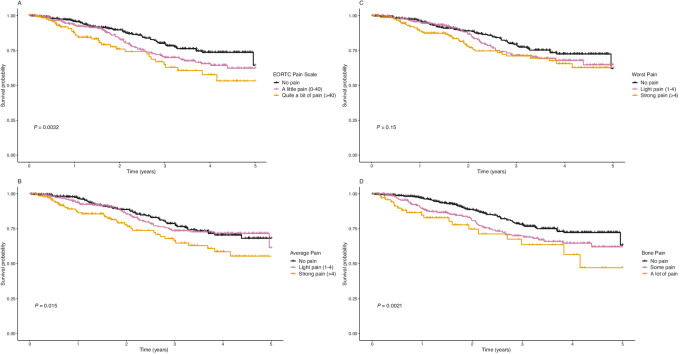
Kaplan–Meier curves for overall survival by pain categories at study enrollment, IRONMAN Registry 2017–2023. **A,** Kaplan–Meier curve for the EORTC pain scale. **B,** Kaplan–Meier curve for the average pain scale. **C,** Kaplan–Meier curve for the worst pain scale. **D,** Kaplan–Meier curve for the bone pain scale.

The 80th percentile survival for participants experiencing the highest amounts of pain at enrollment was substantially lower than the 80th percentile survival for participants experiencing no pain at enrollment for all four pain scales (Fig. 2; Supplementary Table S5). For example, the 80th percentile survival for participants reporting a score of more than 40 on the EORTC pain scale at baseline was 1.60 years (95% CI: 0.99–2.66) compared with 3.01 years (95% CI: 2.50–3.83) for participants with no pain on the EORTC scale at baseline.

### Association Between Pain and All-cause Mortality

For all four pain scales, more frequent or severe pain at study enrollment was associated with an increased risk of all-cause mortality (Table 3). Most notably, compared with participants with no bone pain, participants with a lot of bone pain at baseline had an adjusted HR for death of 2.47 (95% CI: 1.44–4.22). The association between pain and all-cause mortality was stronger for participants with CRPC compared with those with mHSPC and was similar among Black and White participants (Table 4). Results were robust to varying missing data assumptions in the MICE procedure (Supplementary Tables S6–S9).

**TABLE 3:** HRs and 95% CIs for the association between baseline and longitudinal pain scales and death, IRONMAN Registry 2017–2023 (*N* = 879; 137 deaths in White participants, 37 deaths in Black participants)

		Pain at enrollment		Longitudinal pain	
Pain scale	Comparison	Age-only model HR (95% CI)	Fully-adjusted model^a^ HR (95% CI)	Age-only model HR (95% CI)	Fully-adjusted model^a^ HR (95% CI)
EORTC scale	10 points on 0–100 scale	1.12 (1.06–1.17)	1.10 (1.03–1.17)	1.30 (1.20–1.42)	1.29 (1.19–1.40)
Average pain	1 point on 1–10 scale	1.22 (1.10–1.35)	1.19 (1.08–1.32)	1.34 (1.21–1.48)	1.32 (1.20–1.46)
Worst pain	1 point on 1–10 scale	1.17 (1.08–1.26)	1.16 (1.08–1.25)	1.31 (0.90–1.91)^b^	1.31 (1.20–1.43)
Bone pain	Some vs. none	1.62 (1.12–2.33)	1.61 (1.10–2.37)	—^c^	—^c^
Bone pain	A lot vs. none	2.70 (1.65–4.42)	2.47 (1.44–4.22)	—^c^	—^c^

^a^Cox model for pain and death adjusted for potential confounders of disease burden including age at enrollment, first PSA level on-study, Gleason score, disease state at enrollment (mHSPC vs. CRPC), *de novo* metastatic disease at baseline, and sites of metastases at baseline.

^b^SE is inflated as all imputed datasets led to Hessian matrices that were not positive definite for the worst pain scale.

^c^Longitudinal models for bone pain not fit because categorical outcomes are currently incompatible with joint longitudinal survival model capabilities in JM R package.

**TABLE 4:** Stratified analyses for the association between pain scales at enrollment and death, IRONMAN Registry 2017–2023

		mHSPC (*N* = 573)		CRPC (*N* = 306)	
Pain scale	Comparison	Age-only model HR (95% CI)	Fully-adjusted model^a^ HR (95% CI)	Age-only model HR (95% CI)	Fully-adjusted model^a^ HR (95% CI)
EORTC scale	10-points on 0–100 scale	1.08 (1.00–1.17)	1.08 (0.99–1.18)	1.15 (1.06–1.24)	1.12 (1.03–1.22)
Average pain	1 point on 1–10 scale	1.14 (0.99–1.30)	1.12 (0.97–1.30)	1.31 (1.11–1.54)	1.25 (1.06–1.47)
Worst pain	1 point on 1–10 scale	1.12 (1.02–1.24)	1.13 (1.01–1.26)	1.21 (1.07–1.38)	1.18 (1.05–1.32)
Bone pain	Some vs. none	1.02 (0.57–1.83)	0.98 (0.54–1.79)	2.54 (1.69–3.81)	2.43 (1.56–3.79)
Bone pain	A lot vs. none	2.48 (1.11–5.52)	2.22 (0.99–4.98)	3.19 (1.22–8.32)	3.01 (1.00–9.06)
		**White (*N* = 704 total, 137 deaths)**		**Black (*N* = 175 total, 37 deaths)**	
**Pain scale**	**Comparison**	**Age-only model** **HR (95% CI)**	**Fully-adjusted model** **^b^** **HR (95% CI)**	**Age-only model** **HR (95% CI)**	**Fully-adjusted model** **^b^** **HR (95% CI)**
EORTC scale	10 points on 0–100 scale	1.11 (1.04–1.18)	1.10 (1.02–1.18)	1.11 (1.04–1.19)	1.09 (0.98–1.21)
Average pain	1 point on 1–10 scale	1.21 (1.07–1.37)	1.20 (1.04–1.37)	1.25 (1.11–1.40)	1.24 (1.06–1.44)
Worst pain	1 point on 1–10 scale	1.14 (1.04–1.25)	1.14 (1.04–1.25)	1.26 (1.11–1.42)	1.27 (1.05–1.53)
Bone pain	Some vs. none	1.56 (1.05–2.32)	1.53 (1.02–2.29)	1.64 (0.59–4.50)	2.85 (0.73–11.15)
Bone pain	A lot vs. none	2.76 (1.64–4.64)	2.56 (1.47–4.46)	2.61 (0.88–7.80)	3.57 (1.02–12.55)

^a^Cox model for pain and death adjusted for age at enrollment, first PSA level on-study, Gleason score, metastatic status at baseline, *de novo* metastases at baseline, and sites of metastases at baseline.

^b^Cox model for pain and death adjusted for age at enrollment, first PSA level on-study, Gleason score, disease state at enrollment (mHSPC vs. CRPC), metastatic status at baseline, *de novo* metastases at baseline, and sites of metastases at baseline.

For the EORTC pain scale, average pain rating, and worst pain rating, more frequent or severe pain longitudinally was associated with an increased risk of mortality (Table 3). A one-point increase in average pain severity at each timepoint longitudinally was associated with an average 34% higher hazard of mortality (95% CI: 1.21–1.48). In the longitudinal survival models, the association between more frequent or severe pain and risk of death was larger than in the respective survival models for baseline pain.

## Discussion

We found a high prevalence of pain at the time of enrollment in the IRONMAN registry across multiple aspects of pain, including the interference of pain with daily life, average and worst pain ratings, and presence of bone pain. While the majority of participants reported at least some pain on any scale at study enrollment, the mean pain interference level as reported on the EORTC scale in our population was less burdensome than that of the EORTC reference population in 2008 by approximately 10 points on a 100-point scale. (30) This is likely due to the EORTC population including a more representative advanced prostate cancer population in addition to only including individuals who have not yet begun cancer treatment.

In our study population, Black participants reported more pain than White participants across all four pain scales at study enrollment. Though not described in this study, our group previously explored longitudinal pain in this population, finding that pain increased over the first year of follow-up similarly for both Black and White participants (10). As race is a social construct, we believe that the racial disparities we see in pain in our study are a result of structural racism and social determinants of health impacting the experience, reporting, and management of pain. We believe that these factors (including variables like education, employment status, disease characteristics, and type of health center at which the participant receives care) are all mediators of the association between race and pain. Given the incompleteness of data for variables representing social determinants of health in IRONMAN and the lack of collection of information on income and insurance status, we chose to descriptively assess the association between race and pain in this study population. We hope that this study lays the groundwork for future studies that are better equipped for targeted mediation analyses with more complete data on social factors impacting reporting of pain to disentangle mechanisms and potential points for intervention.

One potential mechanism underlying the racial disparities in pain that we see at enrollment could be whether disease characteristics were more severe in Black participants compared with White participants (as is typically the case in the general population of individuals with prostate cancer), where greater disease burden leads to increased pain. Interestingly, we found that Black and White participants had a similar prevalence of high-grade tumors, CRPC, and *de novo* metastatic cancer in our study; only PSA levels at enrollment were significantly elevated in Black participants. It will be important for future studies to investigate the different dimensions of pain in this population to identify additional mechanisms upon which to intervene to improve survivorship, including how patients experience the different dimensions of pain, racial bias in provider acknowledgment of pain and analgesic prescribing patterns, and access to prostate cancer therapies.

Regarding the association between pain and survival, we found that higher pain on all four pain scales at study enrollment was associated with higher mortality after controlling for measures of disease burden and adjusting SEs to account for potential correlation within study site. We also found that worse pain interference, average pain, and worst pain scales longitudinally was associated with higher mortality independent of clinical factors associated with increased disease burden. Our results show similar associations between pain interference and survival compared with previous randomized controlled trials of disease-directed therapies for CRPC or mHSPC in which participants are largely White, well resourced, and without other major medical comorbidities (13–15). As an observational study with eligibility criteria only requiring being diagnosed with mHSPC or CRPC and receiving no more than 90 days of treatment at the time of enrollment, IRONMAN is more inclusive of individuals from different races and socioeconomic backgrounds compared with traditional clinical trials. Though there is still a need to make observational studies like IRONMAN more accessible to more representative patient populations, our study validates these previous clinical trial results in a more real-world population of individuals with advanced prostate cancer in the United States compared with these previous studies.

The biologic mechanisms causing pain are complex and poorly understood, involving the interplay between tumor, bone, inflammatory, and nerve cells. In addition, pain can be caused by the cancer itself or specific cancer therapies and is also influenced by structural racism and individual-level factors (31–36). Because of this, it is challenging to ascertain the mechanism by which pain is associated with a worse prognosis in our study. The IRONMAN registry collects a number of biologic samples that will soon allow a deeper understanding of the biologic drivers of cancer-associated pain, potentially leading to more effective palliative therapies.

There are several potential limitations of this analysis. First, potential unmeasured confounding by prostate cancer therapy could bias our results because lines of therapy in IRONMAN are currently in the process of being extracted. While ADT and ARSIs (the most commonly received therapies in this population) can alleviate pain due to cancer in untreated disease, they have also been associated with exacerbation of osteoarthritic and back pain (37, 38), leading to a downward bias in our HRs. Second, we did not have access to information on prescription and use of analgesics in the IRONMAN population, which is important given their impact on the experience and reporting of pain as well as racial differences in prescriptions of analgesics in patients with cancer (7). As the association between pain and survival is similar for both Black and White participants in our study population, we do not think that racial differences in prescribing patterns of analgesics is greatly impacting our results; however, the overall use of analgesics and their mediating role of the pain-survival relationship in the study population as a whole remains unclear. Third, survivor bias may be inflating our survival estimates as IRONMAN eligibility criteria allows for any diagnosis of advanced prostate cancer as long as there has been no longer than 90 days of systemic cancer-directed treatment at the time of enrollment. As such, it is possible that there are individuals whose pain at diagnosis increases their likelihood to miss follow-up visits or die prior to enrolling in IRONMAN, leading to a stronger true association between pain and all-cause mortality in the overall population of individuals diagnosed with advanced prostate cancer compared with the estimates we see here. Finally, these results may not be generalizable to individuals who choose not to participate in IRONMAN or individuals receiving care at other health centers within or outside of the United States. Centers participating in IRONMAN tend to be highly resourced and located in urban environments; individuals living in more rural areas of the United States or receiving care at urban centers with less clinical trial infrastructure could have different distributions and trajectories of pain and survival.

Our analysis deepens the understanding of racial disparities in pain and survival in individuals with advanced prostate cancer. Black participants experienced substantially higher pain at study enrollment compared with White participants, and more pain is associated with higher mortality independent of covariates related to disease burden. Our analysis highlights the need for further investigation of the experience and management of pain in this population in addition to the biologic drivers of the association between pain and mortality to improve survivorship, particularly for Black individuals experiencing the most severe pain and highest prostate cancer mortality.

## Supplementary Material

Supplementary Table S1 Study sites for IRONMAN participants by race (N = 38 sites)

Supplementary Table S2 Number of participants receiving each therapy category between 3 months prior to enrollment and any time after enrollment stratified by disease state and self-reported race (N=19 therapy categories), N (%)

Supplementary Table S3 Correlation coefficients between baseline pain scale scores

Supplementary Table S4 Reasons for being off-study by self-reported race

Supplementary Table S5 80th percentile survival and 95% confidence intervals for each category of pain at study enrollment

Supplementary Table S6 Baseline EORTC pain scale Cox model results from sensitivity analysis for missing indicator values during MICE procedure

Supplementary Table S7 Baseline average pain scale Cox model results from sensitivity analysis for missing indicator values during MICE procedure

Supplementary Table S8 Baseline worst pain scale Cox model results from sensitivity analysis for missing indicator values during MICE procedure

Supplementary Table S9 Baseline bone pain scale Cox model results from sensitivity analysis for missing indicator values during MICE procedure

Supplementary Figure S1 Longitudinal observation status of questionnaires and reasons for being off-study throughout follow-up

Supplementary Figure S2 Selection of study participants exclusion flowchart

Supplementary Figure S3 Kaplan-Meier survival curve by self-reported race

Supplementary Figure S4 Kaplan-Meier survival curve by disease state at study enrollment

Supplementary Methods S1 Further information guiding the choice of methods and a glossary of technical terms
