# Supplementary material for: Pain and Its Association with Survival for Black and White Individuals with Advanced Prostate Cancer in the United States
Source: Cancer Res Commun. 2024 Jan 8;4(1):55–64. doi: 10.1158/2767-9764.CRC-23-0446 (PMC10773321; doi:10.1158/2767-9764.CRC-23-0446)
Supplement: Supplementary Table S1 — Study sites for IRONMAN participants by race (N = 38 sites) [file crc-23-0446-s01.docx]

**Supplementary Table S1:** Study sites for IRONMAN participants by race (N = 38 sites)

|  | **White (N=704)** | **Black (N=175)** |
| --- | --- | --- |
| Baylor College of Medicine | 6 (0.9%) | 10 (5.7%) |
| Chesapeake Urology Associates | 6 (0.9%) | 5 (2.9%) |
| Columbia University | 4 (0.6%) | 2 (1.1%) |
| Dana-Farber Cancer Institute | 41 (5.8%) | 3 (1.7%) |
| Delnor Cancer Center | 20 (2.8%) | 1 (0.6%) |
| Doylestown Health | 14 (2.0%) | 0 (0%) |
| Duke Cancer Network | 5 (0.7%) | 6 (3.4%) |
| Duke Comprehensive Cancer Center | 55 (7.8%) | 11 (6.3%) |
| Kishwaukee Cancer Center | 1 (0.1%) | 0 (0%) |
| Memorial Sloan Kettering Cancer Center | 65 (9.2%) | 8 (4.6%) |
| Memphis VA Medical Center | 4 (0.6%) | 6 (3.4%) |
| Moffitt Cancer Center | 3 (0.4%) | 0 (0%) |
| Oregon Health and Sciences Cancer Center | 24 (3.4%) | 0 (0%) |
| Ralph H. Johnson VA Medical Center | 10 (1.4%) | 5 (2.9%) |
| Reading Health System | 9 (1.3%) | 2 (1.1%) |
| Robert H. Lurie Comprehensive Cancer Center Northwestern University | 6 (0.9%) | 0 (0%) |
| Roswell Park Cancer Institute | 5 (0.7%) | 1 (0.6%) |
| Sidney Kimmel Comprehensive Cancer Center | 7 (1.0%) | 6 (3.4%) |
| Thomas Jefferson University | 3 (0.4%) | 1 (0.6%) |
| Tulane University | 53 (7.5%) | 8 (4.6%) |
| University of Alabama-Birmingham | 4 (0.6%) | 3 (1.7%) |
| University of California Los Angeles | 7 (1.0%) | 0 (0%) |
| University of California San Diego | 31 (4.4%) | 2 (1.1%) |
| University of Chicago | 20 (2.8%) | 12 (6.9%) |
| University of Illinois at Chicago | 4 (0.6%) | 1 (0.6%) |
| University of Michigan | 27 (3.8%) | 4 (2.3%) |
| University of Mississippi Medical Center | 3 (0.4%) | 5 (2.9%) |
| University of North Carolina | 31 (4.4%) | 8 (4.6%) |
| University of Virginia | 113 (16.1%) | 15 (8.6%) |
| University of Washington | 39 (5.5%) | 1 (0.6%) |
| University of Wisconsin | 8 (1.1%) | 0 (0%) |
| Warrenville Cancer Center | 1 (0.1%) | 0 (0%) |
| Wayne St. University Karmanos Cancer Institute | 24 (3.4%) | 28 (16.0%) |
| Weill Cornell Medical Center | 44 (6.3%) | 4 (2.3%) |
| Winship Cancer Institute Emory University | 7 (1.0%) | 9 (5.1%) |
| Durham VA Medical Center | 0 (0%) | 5 (2.9%) |
| Fox Chase Cancer Center - Temple Health | 0 (0%) | 1 (0.6%) |
| New York-Presbyterian Brooklyn Methodist Hospital | 0 (0%) | 2 (1.1%) |
