# Supplementary material for: Pain and Its Association with Survival for Black and White Individuals with Advanced Prostate Cancer in the United States
Source: Cancer Res Commun. 2024 Jan 8;4(1):55–64. doi: 10.1158/2767-9764.CRC-23-0446 (PMC10773321; doi:10.1158/2767-9764.CRC-23-0446)
Supplement: Supplementary Table S2 — Number of participants receiving each therapy category between 3 months prior to enrollment and any time after enrollment stratified by disease state and self-reported race (N=19 therapy categories), N (%) [file crc-23-0446-s02.docx]

**Supplementary Table S2:** Number of participants receiving each therapy category between 3 months prior to enrollment and any time after enrollment stratified by disease state and self-reported race (N=19 therapy categories), N (%)

|  | **CRPC** | | **mHSPC** | |
| --- | --- | --- | --- | --- |
|  | **White (N=241)** | **Black (N=65)** | **White (N=463)** | **Black (N=110)** |
| ADT | 221 (91.7) | 54 (83.1) | 417 (90.0) | 90 (81.8) |
| AR Signaling Inhibitor | 184 (76.3) | 46 (70.8) | 304 (65.7) | 57 (51.8) |
| Chemotherapy | 61 (25.3) | 19 (29.2) | 109 (23.5) | 25 (22.7) |
| Immunotherapy | 37 (15.4) | 11 (16.9) | 23 (5.0) | 3 (2.7) |
| Radiopharmaceuticals | 28 (11.6) | 10 (15.4) | 16 (3.5) | 3 (2.7) |
| PARP | 14 (5.8) | 3 (4.6) | 6 (1.3) | 0 (0) |
| Multi-kinase Inhibitor | 0 (0) | 4 (6.2) | 0 (0) | 2 (1.8) |
| Immunotherapy/Placebo | 3 (1.2) | 0 (0) | 2 (0.4) | 0 (0) |
| mTOR kinase/DNA-PK Inhibitor | 3 (1.2) | 2 (3.1) | 0 (0) | 0 (0) |
| CDK Inhibitor | 1 (0.4) | 0 (0) | 1 (0.2) | 0 (0) |
| TGF-ßRI kinase Inhibitor | 2 (0.8) | 0 (0) | 0 (0) | 0 (0) |
| CDK Inhibitor/Placebo | 0 (0) | 0 (0) | 1 (0.2) | 0 (0) |
| EZH2 Inhibitor | 1 (0.4) | 0 (0) | 0 (0) | 0 (0) |
| GR Antagonist | 0 (0) | 0 (0) | 1 (0.2) | 0 (0) |
| PARP/Placebo | 1 (0.4) | 0 (0) | 0 (0) | 0 (0) |
| PI3K Inhibitor | 0 (0) | 0 (0) | 1 (0.2) | 0 (0) |
| PLK1 Inhibitor | 0 (0) | 0 (0) | 1 (0.2) | 0 (0) |
| SHP2 Inhibitor | 0 (0) | 0 (0) | 1 (0.2) | 0 (0) |
| α/β Tubulin Inhibitor | 1 (0.4) | 0 (0) | 0 (0) | 0 (0) |

Note: participants can fall into more than one group and often are receiving multiple of these therapy categories at the same time
