# Supplementary material for: Pain and Its Association with Survival for Black and White Individuals with Advanced Prostate Cancer in the United States
Source: Cancer Res Commun. 2024 Jan 8;4(1):55–64. doi: 10.1158/2767-9764.CRC-23-0446 (PMC10773321; doi:10.1158/2767-9764.CRC-23-0446)
Supplement: Supplementary Table S3 — Correlation coefficients between baseline pain scale scores [file crc-23-0446-s03.docx]

**Supplementary Table S3:** Correlation coefficients between baseline pain scale scores

|  | **EORTC Pain**  **Scale** | **Average**  **Pain** | **Worst**  **Pain** | **Bone**  **Pain** |
| --- | --- | --- | --- | --- |
| **EORTC Pain Scale** | 1.00 | 0.75 | 0.80 | 0.56 |
| **Average Pain** | 0.75 | 1.00 | 0.87 | 0.59 |
| **Worst Pain** | 0.80 | 0.87 | 1.00 | 0.58 |
| **Bone Pain** | 0.56 | 0.59 | 0.58 | 1.00 |
