# Supplementary material for: Pain and Its Association with Survival for Black and White Individuals with Advanced Prostate Cancer in the United States
Source: Cancer Res Commun. 2024 Jan 8;4(1):55–64. doi: 10.1158/2767-9764.CRC-23-0446 (PMC10773321; doi:10.1158/2767-9764.CRC-23-0446)
Supplement: Supplementary Table S4 — Reasons for being off-study by self-reported race [file crc-23-0446-s04.docx]

**Supplementary Table S4:** Reasons for being off-study by self-reported race

| **Off-study reason** | **White participants (N=704)** | **Black participants (N=175)** |
| --- | --- | --- |
| Death | 137 (19.5%) | 37 (21.4%) |
| Withdrawal by participant | 59 (8.4%) | 13 (7.4%) |
| Lost to follow-up | 31 (4.4%) | 10 (5.7%) |
| Study termination | 26 (3.7%) | 5 (2.9%) |
| Clinical deterioration | 22 (3.1%) | 9 (5.1%) |
