# Supplementary material for: Pain and Its Association with Survival for Black and White Individuals with Advanced Prostate Cancer in the United States
Source: Cancer Res Commun. 2024 Jan 8;4(1):55–64. doi: 10.1158/2767-9764.CRC-23-0446 (PMC10773321; doi:10.1158/2767-9764.CRC-23-0446)
Supplement: Supplementary Table S5 — 80th percentile survival and 95% confidence intervals for each category of pain at study enrollment [file crc-23-0446-s05.docx]

**Supplementary Table S5**: 80^th^ percentile survival and 95% confidence intervals for each category of pain at study enrollment

| **Pain scale** | **No pain** | **A little pain** | **Quite a bit of pain** | **p-value** |
| --- | --- | --- | --- | --- |
| EORTC pain scale | 3.01 (2.50, 3.83) | 2.19 (1.90, 2.57) | 1.60 (0.99, 2.66) | 0.0032 |
| Average pain rating | 2.81 (2.41, 3.69) | 2.40 (2.09, 3.32) | 1.82 (1.08, 2.70) | 0.015 |
| Worst pain rating | 2.84 (2.57, 3.83) | 2.34 (2.06, 2.90) | 1.93 (1.60, 3.29) | 0.15 |
| Bone pain presence | 2.81 (2.50, 3.69) | 2.08 (1.66, 2.61) | 1.60 (0.67, 3.83) | 0.0021 |

Note: p-value reported is from log-rank test of Kaplan-Meier curves
