# Supplementary material for: Pain and Its Association with Survival for Black and White Individuals with Advanced Prostate Cancer in the United States
Source: Cancer Res Commun. 2024 Jan 8;4(1):55–64. doi: 10.1158/2767-9764.CRC-23-0446 (PMC10773321; doi:10.1158/2767-9764.CRC-23-0446)
Supplement: Supplementary Table S6 — Baseline EORTC pain scale Cox model results from sensitivity analysis for missing indicator values during MICE procedure [file crc-23-0446-s06.docx]

**Supplementary Table S6**: Baseline EORTC pain scale Cox model results from sensitivity analysis for missing indicator values during MICE procedure (see Supplementary Methods S1 for more information)

| **Missing Indicator Value** | **HR (95% CI)** |
| --- | --- |
| -250 | 1.104 (1.034, 1.173) |
| -200 | 1.104 (1.034, 1.172) |
| -150 | 1.102 (1.033, 1.170) |
| -100 | 1.102 (1.032, 1.171) |

EORTC pain scale ranged from 0-100. Missing indicators for other scales during the imputation procedure were -25 (average and worst pain) and -10 (bone pain).
