# Supplementary material for: Pain and Its Association with Survival for Black and White Individuals with Advanced Prostate Cancer in the United States
Source: Cancer Res Commun. 2024 Jan 8;4(1):55–64. doi: 10.1158/2767-9764.CRC-23-0446 (PMC10773321; doi:10.1158/2767-9764.CRC-23-0446)
Supplement: Supplementary Table S8 — Baseline worst pain scale Cox model results from sensitivity analysis for missing indicator values during MICE procedure [file crc-23-0446-s08.docx]

**Supplementary Table S8**: Baseline worst pain scale Cox model results from sensitivity analysis for missing indicator values during MICE procedure (see Supplementary Methods S1 for more information)

| **Missing Indicator Value** | **HR (95% CI)** |
| --- | --- |
| -25 | 1.162 (1.078, 1.252) |
| -20 | 1.161 (1.083, 1.245) |
| -15 | 1.165 (1.084, 1.252) |
| -10 | 1.161 (1.083, 1.244) |

Worst pain scale ranged from 1-10. Missing indicators for other scales during the imputation procedure were -250 (EORTC pain scale), -25 (average pain), and -10 (bone pain).
