# Supplementary material for: Pain and Its Association with Survival for Black and White Individuals with Advanced Prostate Cancer in the United States
Source: Cancer Res Commun. 2024 Jan 8;4(1):55–64. doi: 10.1158/2767-9764.CRC-23-0446 (PMC10773321; doi:10.1158/2767-9764.CRC-23-0446)
Supplement: Supplementary Table S9 — Baseline bone pain scale Cox model results from sensitivity analysis for missing indicator values during MICE procedure [file crc-23-0446-s09.docx]

**Supplementary Table S9**: Baseline bone pain scale Cox model results from sensitivity analysis for missing indicator values during MICE procedure (see Supplementary Methods S1 for more information)

| **Missing Indicator Value** | **HR (95% CI) – some vs. none** | **HR (95% CI) – a lot vs. none** |
| --- | --- | --- |
| -10 | 1.608 (1.097, 2.374) | 2.472 (1.440, 4.216) |
| -9 | 1.611 (1.101, 2.373) | 2.475 (1.439, 4.216) |
| -8 | 1.609 (1.108, 2.366) | 2.477 (1.435, 4.218) |
| -7 | 1.613 (1.118, 2.372) | 2.488 (1.450, 4.228) |

Bone pain scale ranged from 0-4. Missing indicators for other scales during the imputation procedure were -250 (EORTC pain scale) and -25 (average and worst pain).
