# Supplementary material for: Pain and Its Association with Survival for Black and White Individuals with Advanced Prostate Cancer in the United States
Source: Cancer Res Commun. 2024 Jan 8;4(1):55–64. doi: 10.1158/2767-9764.CRC-23-0446 (PMC10773321; doi:10.1158/2767-9764.CRC-23-0446)
Supplement: Supplementary Figure S1 — Longitudinal observation status of questionnaires and reasons for being off-study throughout follow-up [file crc-23-0446-s10.pdf]

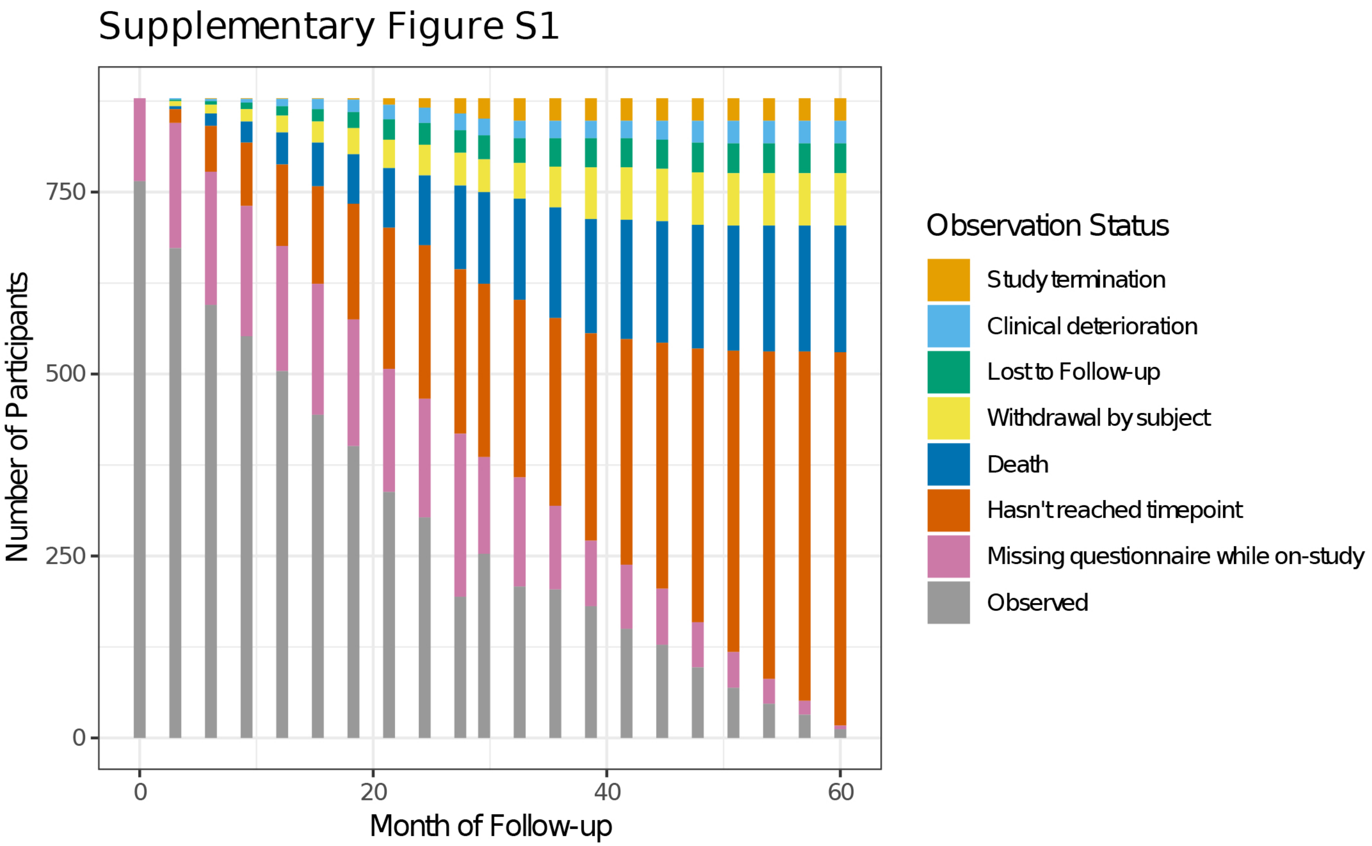

Supplementary Figure S1 shows shows the proportion of questionnaires falling under each observation status at each 3-month point of follow-up.
