# Supplementary material for: Pain and Its Association with Survival for Black and White Individuals with Advanced Prostate Cancer in the United States
Source: Cancer Res Commun. 2024 Jan 8;4(1):55–64. doi: 10.1158/2767-9764.CRC-23-0446 (PMC10773321; doi:10.1158/2767-9764.CRC-23-0446)
Supplement: Supplementary Figure S2 — Selection of study participants exclusion flowchart [file crc-23-0446-s11.pdf]

Supplementary Figure S2

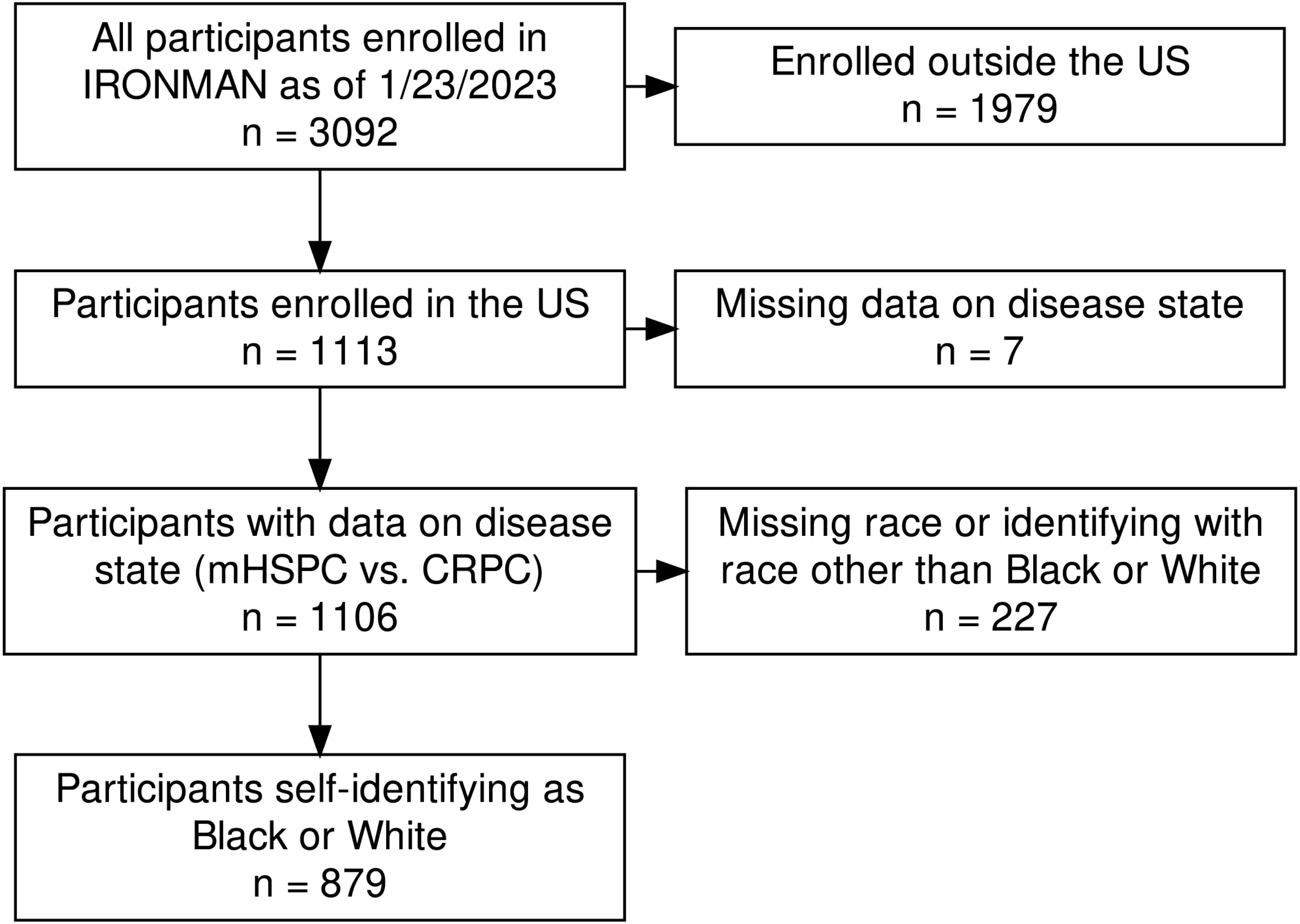

Supplementary Figure S2 shows the exclusion criteria used to define the study population for this analysis.
