# Supplementary material for: Pain and Its Association with Survival for Black and White Individuals with Advanced Prostate Cancer in the United States
Source: Cancer Res Commun. 2024 Jan 8;4(1):55–64. doi: 10.1158/2767-9764.CRC-23-0446 (PMC10773321; doi:10.1158/2767-9764.CRC-23-0446)
Supplement: Supplementary Figure S3 — Kaplan-Meier survival curve by self-reported race [file crc-23-0446-s12.pdf]

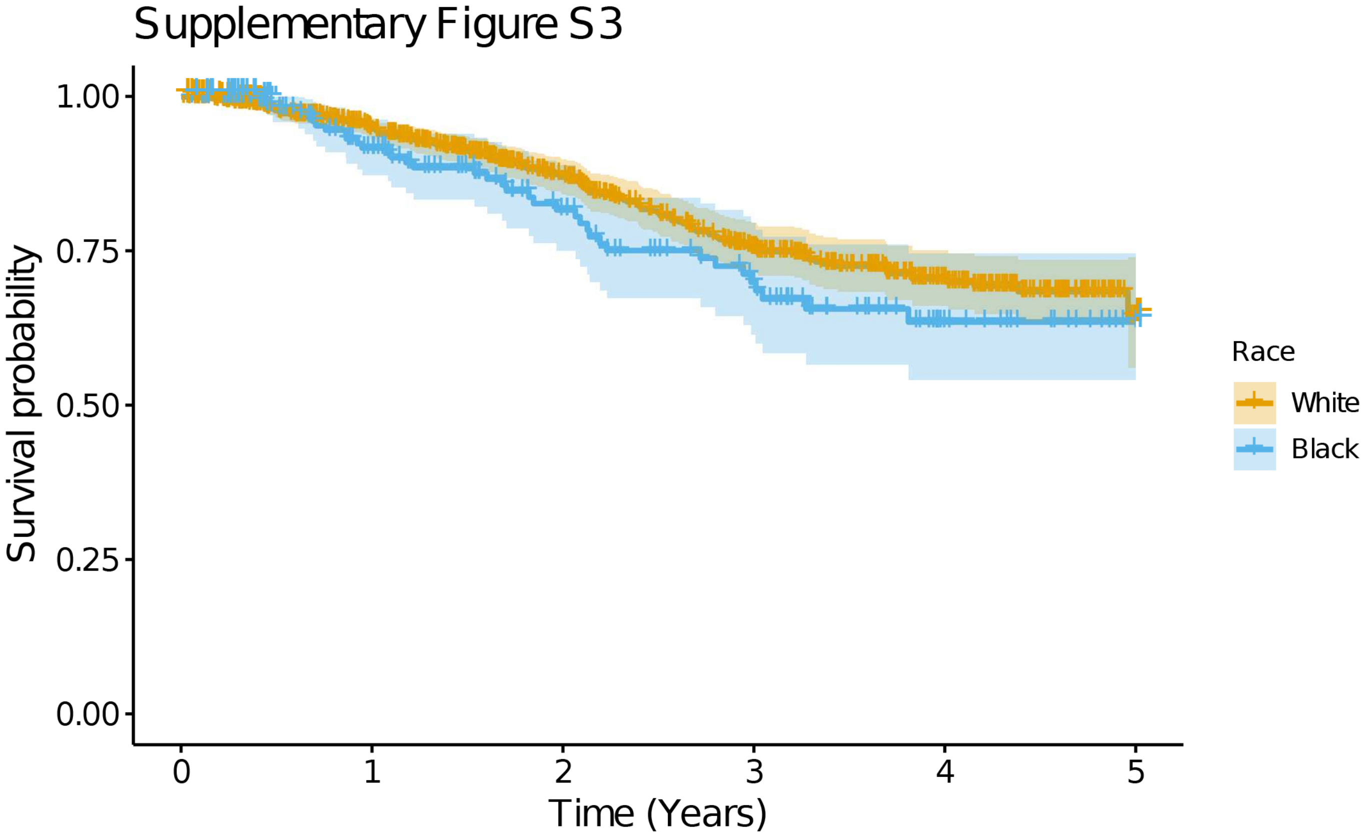


**Supplementary Figure S3 shows the Kaplan-Meier curve for survival stratified by race.**
