# Supplementary material for: Pain and Its Association with Survival for Black and White Individuals with Advanced Prostate Cancer in the United States
Source: Cancer Res Commun. 2024 Jan 8;4(1):55–64. doi: 10.1158/2767-9764.CRC-23-0446 (PMC10773321; doi:10.1158/2767-9764.CRC-23-0446)
Supplement: Supplementary Methods S1 — Further information guiding the choice of methods and a glossary of technical terms [file crc-23-0446-s14.docx]

**Supplementary Methods S1**

This section describes our exploration of missing data in two parts: 1) missing individual variables on completed questionnaires, and 2) missing entire questionnaires, particularly an exploration of censoring as IRONMAN is ongoing.

*Missing individual variables on completed questionnaires*

We began by exploring missing data on questionnaires that were completed by the participants. Throughout follow-up, there were 6150 total completed questionnaires for the EORTC, average pain, and worst pain questions, and 3331 total completed questionnaires for bone pain based on the frequency at which the questions were asked and the number of participants remaining on-study over time.

Missingness of pain questions on completed questionnaires was small (~3% for the bone pain question to less than 1% for the EORTC questions). We used multiple imputation by chained equations to create imputed datasets for the missing pain questions. As we were interested in the scale score for the EORTC pain questions rather than the individual questions, we calculated the EORTC scale score where both questions were complete and imputed the scale scores.(1) We began by making a long dataset where each row represented a single timepoint for one individual. As we only wanted to impute the missing pain questions on completed questionnaires, we set the pain scales on missing full questionnaires to a missing indicator value (-250 for the EORTC scale, -25 for the average and worst pain scales, and -10 for the bone pain question). The missing indicator values needed to be numeric and also outside of the range of each scale so as to not bias the results. We then converted to a wide dataset with one row per individual to preserve clustering by participant during the imputation process.

MICE was conducted on the wide dataset using the following variables(2): participant ID, race, marital status, highest education attained, employment status, military status, metastatic status at baseline, study site ID, disease state at enrollment, age, PSA, Gleason score, de novo metastatic disease, location of metastases, type of health center at which the participant received care, year of enrollment, death status, and time on study. We used the MICE method of classification and regression trees and constrained the outcome variable imputations to be between the possible bounds of the range for each scale (0-100 for the EORTC scale, 1-10 for average and worst pain, and 0-4 for bone pain). We created 10 imputed datasets using 10 iterations for each imputation to stabilize. After the MICE procedure, we had full pain scale data for completed questionnaires and full covariate data for all participants. We then converted back to a long dataset and returned the numeric indicator values back to missing.

We conducted sensitivity analyses using different values for the missing indicator to determine how robust our results of the longitudinal analysis were to the chosen value. The results of these sensitivity analyses for each pain scale in the baseline Cox model are shown in **Supplementary Tables S6-S9.** The most negative values were chosen for the final analysis as they were the furthest from the scale of the pain questions.

*Missing entire questionnaires*

To assess missingness of entire questionnaires, we classified questionnaires into four groups: 1) completed, 2) missing while on-study, 3) censored due to death, withdrawal, etc, and 4) censored due to not accruing enough person-time on-study to complete the questionnaire (as IRONMAN enrollment is ongoing).

To visualize the longitudinal missingness in the data, we created **Supplementary Figure S1** showing the observation status of each questionnaire/reason for being off-study for each participant over five years of follow-up. 345 participants (39%) were off-study during follow-up.

As IRONMAN enrollment is ongoing, there have been numerous protocol changes, and a global pandemic occurred in the middle of study enrollment, we wanted to assess the role of censoring in these data. To do this, we investigated the distribution of censoring times by year of enrollment (excluding participants who died). We found that late censoring occurs in participants who were enrolled early on, and early censoring was primarily among participants who were recently enrolled and hadn’t yet accrued person-time sufficient for long-term follow-up. From this, we determined that censoring likely isn’t impacting our results substantially unless there are substantial cohort differences by enrollment year.

To explore potential cohort differences by enrollment year, we assessed differences in demographic/clinical variables, study site, and baseline pain scores by enrollment year. We categorized enrollment year as a binary variable with one level representing enrollment in the first half of the study (June 2017 – June 2020), and the other level representing enrollment in the second half of the study (July 2020 – February 2023). We found statistically significant differences in disease state at enrollment (higher proportion of mHSPC during the second half of enrollment), highest education level at baseline (higher education during the second half of enrollment), and type of health center (higher proportion of participants receiving care at VA and community hospitals during the second half of enrollment).

We also investigated differences in site accrual by enrollment year. NCI-designated health centers tended to enroll fewer participants in the second half of the study (likely due to clinical trial enrollment changes during the COVID pandemic) and sites added as participating centers later (eg the Ralph H. Johnson VA Medical Center) only enrolled participants in the second half of the study.

Finally, we investigated differences in baseline pain scores by enrollment year. No statistically significant differences were noted in pain questions at study enrollment based on the enrollment year of the participant.

Taken together, we believe that there are likely some cohort differences depending on year of enrollment that could affect our results. As such, we stratified the baseline hazard of our Cox proportional hazards in both the baseline and longitudinal settings by year of enrollment (as a categorical variable from 1 to 6 representing each year of enrollment).

**References**

1. Fayers PM, Aaronson NK, Bjordal K, Groenvold M, Curran D, et al. The EORTC QLQ-C30 Scoring Manual (3^rd^ Edition). Published by: European Organisation for Research and Treatment of Cancer, Brussels 2001.

2. Azur MJ, Stuart EA, Frangakis C, Leaf PJ. Multiple imputation by chained equations: what is it and how does it work? Int J Methods Psychiatr Res. 2011;20(1):40–9. PMCID: PMC3074241.

**Glossary of technical terms**

**Advanced prostate cancer**: cancer that has spread outside of the prostate gland (mHSPC) and/or cancer that is resistant to hormone therapy (CRPC).

**Castration resistant prostate cancer (CRPC)**: cancer that is either only in the prostate gland or has spread throughout the body that does not respond to hormone therapy. Newer and more aggressive treatment options are typically used.

**Cox proportional hazards model:** a method used to determine the relationship between a variable and the risk of death after accounting for other variables that might bias the results

**Joint longitudinal survival models:** a method used to determine the relationship between the longitudinal trend of a variable and the risk of death after accounting for other variables that might bias the results

**Kaplan-Meier estimates:** a method used to create a graph that shows the survival probability for individuals in a group over time

**Linear mixed effects model**: a mathematical equation that allows researchers to determine the relationship between a variable of interest and an outcome of interest. This type of equation is used for continuous outcomes and allows us to group data over time within individuals (ie one person filling out a questionnaire at 5 timepoints) and group individuals within sites at which they receive care.

**Localized prostate cancer**: cancer that is only present inside the prostate gland and has not spread to other parts of the body. Typically treated with surgery and/or radiation, sometimes also treated with hormone therapy.

**Metastatic hormone-sensitive prostate cancer (mHSPC)**: cancer that has spread outside of the prostate gland but is still responsive to hormone therapy as a treatment.

**Multiple imputation by chained equations (MICE):** a way to fill in missing variables in a dataset by comparing people in the dataset. If an individual in a dataset is missing data on age, for example, MICE would find someone else in the dataset who looks similar to that person (ie a person of the same gender with the same disease state, education level, marital status, etc) and fill in the first missing person’s data with the second person’s age.

**Non-monotone missingness:** a pattern of missingness in which a study participant completes a questionnaire at one timepoint, misses the next timepoint, and then comes back to fill in the questionnaire at the third timepoint.

**Patient-reported outcome measures**: questions asked to patients about their quality of life including questions about pain, fatigue, emotional functioning, etc

**Prospective cohort**: a type of study that enrolls a large number of people in a defined group and follows them over time
